# Involvement and engagement of seldom-heard communities in big data research.

**DOI:** 10.23889/ijpds.v7i3.1891

**Published:** 2022-08-25

**Authors:** Piotr Teodorowski, Saiqa Ahmed, Naheed Tahir, Kate Fleming, Sarah Rodgers, Lucy Frith

**Affiliations:** 1 University of Liverpool; 2 ARC NWC Public Advisor; 3 NHS Digital; 4 University of Manchester

## Objectives

Public involvement and engagement have been growing within big data research. However, seldom heard voices such as migrant and ethnic minorities communities are often underrepresented. This study explored how Polish and South Asian communities in the United Kingdom could be better included in public involvement and engagement activities.

## Approach

We conducted semi-structured interviews with Polish (n=20) and South Asians (n=20) to elicit their views on big data research, public involvement and engagement. We focused on Polish and South Asian communities as they represent some of the United Kingdom’s largest migrant and ethnic minority groups. Data were analysed using inductive thematic analysis. Public advisors were involved in the analysis. They and one of the researchers come from ethnic minority and offered insider insight into participants' perspectives and thus allowing us to unpick the complexity of experiences and backgrounds.

## Results

The majority of participants were willing to become involved or engaged in big data research. However, we found there were multiple barriers to involvement, these included: language (especially for those for whom English is the second language); use of jargon by researchers; time restrictions and unfamiliarity with big data or public involvement. Some participants questioned how much migrants could be involved when they were only in the United Kingdom on a temporary basis. The participants made recommendations for how researchers can mitigate these barriers. Awareness-raising activities would allow people to expand their understanding and build their confidence when speaking about big data research in a second language. Participants spoke of the need for researchers to work more closely with local communities, especially with local gatekeepers.

## Conclusion

The results indicate that there is no ‘right’ way to involve and engage seldom heard communities around big data research. Researchers need to engage with communities, establish trust and develop a long-lasting relationships. These partnerships should move beyond single projects and aim to benefit both researchers and seldom heard communities.

